# lncRNA SNHG15 Induced by SOX12 Promotes the Tumorigenic Properties and Chemoresistance in Cervical Cancer via the miR-4735-3p/HIF1a Pathway

**DOI:** 10.1155/2022/8548461

**Published:** 2022-01-12

**Authors:** Jiang Yang, Mei Yang, Huabing Lv, Min Zhou, Xiaogang Mao, Xiaomin Qin, Ying Xu, Lin Li, Hui Xing

**Affiliations:** Department of Obstetrics and Gynecology, Xiangyang Central Hospital, Affiliated Hospital of Hubei University of Arts and Science, Xiangyang, Hubei, China

## Abstract

Cervical cancer (CC) is one of the most common malignancies in females, with high prevalence and mortality globally. Despite advances in diagnosis and therapeutic strategies developed in recent years, CC is still a major health burden worldwide. The molecular mechanisms underlying the development of CC need to be understood. In this study, we aimed to demonstrate the role of lncRNA SNHG15 in CC progression. Using qRT-PCR, we determined that lncRNA SNHG15 is highly expressed in CC tumor tissues and cells. lncRNA SNHG15 knockdown also reduces the tumorigenic properties of CC *in vitro*, as determined using the MTT, EdU, flow cytometry, and transwell assays. Using bioinformatics analysis, RNA pull-down, ChIP, and luciferase reporter assays, we verified the molecular mechanisms of lncRNA SNHG15 in CC progression and found that lncRNA SNHG15 expression in CC cells is transcriptionally regulated by SOX12; moreover, lncRNA SNHG15 promotes CC progression via the miR-4735-3p/HIF1a axis. This study can provide a potential target for CC diagnosis or therapeutic strategies in the future.

## 1. Introduction

Cervical cancer (CC) ranks next to breast cancer as the most common malignancy and is the third leading cause of cancer-related deaths in females globally [1]. The health burden associated with CC is more severe in developing countries [2], particularly in low-income countries, than in developed countries [2]. Multiple risk factors contribute to the initiation and progression of CC, such as viral infection, genetic influences, and human papillomavirus (HR-HPV) genotype infection [3, 4]. Despite improvement in clinical intervention strategies, such as the global application of standard vaccination, surgical resection innovation, and periodic cancer screening in recent decades, the outcomes of patients with CC remain poor. Therefore, the molecular mechanisms underlying CC development need to be understood, and novel therapeutic targets for the prevention and treatment of CC have to be urgently developed.

Long noncoding RNAs (lncRNAs) are newly discovered no-coding RNAs longer 200 nt [5]. Emerging evidence has elucidated the crucial role of lncRNAs in various diseases, including cancers. lncRNA NBR2 suppresses tumorigenesis in hepatocellular cancer via modulating autophagy level [6]. lncRNA GATA3-AS1 regulates triple-negative breast cancer by facilitating tumorigenesis and immune escape phenomena [7]. lncRNA HOTAIR facilitates exosome secretion in hepatocellular cancer by regulating RAB35 and SNAP23 [8]. lncRNA ASB16-AS1 plays an oncogenic role in renal cell carcinoma progression by acting as a ceRNA for miR-185-5p/miR-214-3p [9]. The lncRNA small nucleolar RNA host gene 15 (lncRNA SNHG15) is located on chromosome 7p13 and was first elucidated in a study of cellular stress responses [10, 11]. The function of lncRNA SNHG15 in various cancers has also been investigated, including breast cancer, colorectal cancer, gastric cancer, hepatocellular cancer, and lung cancer [12–16]. Meanwhile, whether lncRNA SNHG15 participates in CC progression remains unclear.

In this study, we aimed to demonstrate the role of lncRNA SNHG15 in CC progression. We found that lncRNA SNHG15 was abundantly expressed in CC tumors and cells. lncRNA SNHG15 knockdown significantly attenuated tumorigenesis in CC *in vitro* and *in vivo*, as determined using the MTT, EdU, flow cytometry, and transwell assays, in addition to animal experiments. Further, our study revealed that lncRNA SNHG15 influenced the chemoresistance of CC cells to cisplatin. We then investigated the molecular mechanisms of lncRNA SNHG15 in CC cells by conducting bioinformatics analysis, RNA pull-down, ChIP, and luciferase reporter assay. It was found that lncRNA SNHG15 expression in CC cells was transcriptionally regulated by SRY-Box Transcription Factor 12 (SOX12). Further, we found that lncRNA SNHG15 upregulated HIF1a expression to regulate tumorigenesis in CC by sponging miR-4735-3p. Thus, our study elucidated the effect of SOX12/lncRNA SNHG15/miR-4735-3p/HIF1a axis on the biological features of CC.

## 2. Materials and Methods

### 2.1. Clinical Samples

A total of twenty-eight pairs of CC tumor tissues and comparative normal tissues were collected from Xiangyang Central Hospital within the period 2019/06 to 2020/06. All CC tissues were immediately stored in −80°C liquid nitrogen until the experiment was conducted. Informed consent was obtained from each patient. All CC tissues were confirmed by two pathologists, independently. This study was performed following the principles of the Declaration of Helsinki and was approved by the ethical committee of Xiangyang Central Hospital.

### 2.2. Cell Culture and Transfection

All cervical cancer cell lines (SiHa, HeLa, Caski, C-33A, and MS751) and normal cell line HEKn were commercially obtained from the Committee on Type Culture Collection of the Chinese Academy of Sciences (Shanghai, China). Cells were cultured using Dulbecco's Modified Eagle's Medium (DMEM; Invitrogen, USA) supplied with 10% fetal bovine serum (FBS) at 37°C in a humidified 5% CO_2_ environment.

All siRNAs targeting lncRNA SNHG15 were synthesized and obtained from Sigma-Aldrich (USA). Moreover, miRNA mimics, inhibitors, and normal controls were procured from GenePharma (Shanghai, China). The pcDNA3.1 (+) vector (GenePharma, Shanghai) was applied to generate overexpression vectors. The siRNA sequences are as follows: si-NC: F: 5′-CAGUCGCGUUUGCGACUGGC-3′ and R: 5′-GCCAGUCGCAAACGCGACUG-3′, si-SNHG15#1: F: 5′-CCUUGAGUCUCAUGUUCAA-3′ and R: 5′-UUGAACAUGAGACUCAAGG-3′, si-SNHG15#2: F: 5′-GAGCUUACUGUCACAGCAA-3′ and R: 5′-UUGCUGUGACAGUAAGCUC-3′. A Lipofectamine 3000 (Invitrogen) was applied to conduct transfections.

### 2.3. RNA Extraction and qRT-PCR

All RNAs were collected from tissues and cells by using the TRIzol Reagent (Thermo Fisher Scientific) in accordance with the manufacturer protocols. Reverse-transcription was performed using the SuperScript™ III First-Strand Synthesis System (Thermo Fisher Scientific). The expression level of the indicated gene was measured using the SYBR Green Real-Time PCR Master Mixes (Thermo Fisher Scientific) on the ABI Prism 700 thermal cycler (Applied Biosystems, Foster City, CA). U6 and GAPDH were used as internal controls. The relative gene expression was calculated using the 2-*ΔΔ*Ct method. The primers applied in this study are as follows: lncRNA SNHG15: F: GCTGAGGTGACGGTCTCAAA and R: GCCTCCCAGTTTCATGGACA, miR-4735-3p: F: 5′-GGTAGCTGAGAACATTACAG-3′ and R: 5′-CTATTCTGGAACATCAAGCC-3′, HIF1a: F: 5′-TGTGAACCCATTCCTCATCCA-3′ and R: 5′-GGCTCATAACCCATCAACTCA-3′, SOX12: F: 5′-CGCGATGGTGCAGCAGCG-3′ and R: 5′-GCCACTGGTCCATGATCTTC-3′, U6: F: TCCGATCGTGAAGCGTTC and R: GTGCAGGGTCCGAGGT, GAPDH: F: CGGAGTCAACGGATTTGGTCGTAT and R: AGCCTTCTCCATGGTGGTGAAGAC.

### 2.4. Western Blot

All proteins were isolated from tissues and cells by using the RIPA Buffer (Solarbio, R0020). Protein samples were maintained by SDS-PAGE using 8%-12% gels (Beyotime, P0012A). Subsequently, protein samples were then electronically transferred onto a polyvinylidene difluoride membrane (0.45 *μ*m) (Millipore, IPVH00010) and then blocked using 5% skimmed milk. Next, primary antibodies were subsequently used to incubate with membranes at 4°C for 12 hours, followed by incubation with secondary antibodies at room temperature for 2 hours. Results were visualized using the UVP ChemiDoc-It Imaging System (UVP, CA, USA). The antibodies used in this study were as follows: HIF1a (1 : 1000; ab237544; Abcam) and GAPDH (1 : 1000; # 5174S; CST).

### 2.5. Cell Proliferation Detection

*3-(4,5-Dimethylthiazol-2-yl)-2,5-diphenyltetrazolium bromide (MTT) assay*. Cells were cultured in a 96 well-plate for proliferation detection. The culture medium was added sequentially with 10 *μ*L MTT (Invitrogen) for 24, 48, and 72 h. Formazan was dissolved using DMSO (Sigma, USA). After 4 h, the absorbance of each well was measured on a plate reader at 570 nm. The experiment was conducted in triplicate.

*5-Ethynyl-2*′*-deoxyuridine (EdU) experiment*. The experimental cells were first cultured in a 96-well plate after transfection. The EdU kit (KeyGEN BioTECH) was used to stain cells in the well in accordance with instructions provided by the manufacturer. The results (EdU-positive cells) were recorded by a fluorescence microscopy.

### 2.6. Cell Apoptosis Detection

The apoptosis rate of infected CC cells was detected by flow cytometry analysis. The infected CC cells were stained using an Annexin V-fluorescein isothiocyanate (FITC)/propidium iodide (PI) kit (BD Biosciences, USA), and the results were calculated with a flow cytometer using the CellQuest software version 0.9.3.1 (BD Biosciences, USA).

### 2.7. Cell Migration Detection

The migratory ability of infected CC cells was detected using the transwell assay. In summary, the infected CC cells were seeded in the upper chambers (Costar, USA) supplied with the serum-free RPMI-1640 medium, and the lower chambers without cells were supplied with full medium. After incubation for one day, the migrated cells were fixed with 4% paraformaldehyde and then stained with crystal violet. The results were recorded by a microscopy.

### 2.8. Murine Xenograft Assay

SiHa and HeLa cells stably transfected with si-SHNG15#1, si-SHNG15#2, or si-NC were injected into mice aged 4 weeks at a density of 1 × 10^6^ cells. The dimensions of the tumors were determined, and their weights were recorded after injection for 28 d. The animal procedures conducted in this study complied with the guidelines set by the ethical committee of Xiangyang Central Hospital.

### 2.9. RNA Pull-down Assay

SiHa and HeLa cells were infected using 50 nM biotinylated probes, which were synthesized and procured from GenePharma (Shanghai, China). After 2 d, the M-280 streptavidin magnetic beads (Sigma-Aldrich, USA) were used to incubate with the cell lysate at 4°C for 3 h. The beads were then washed, and the bound RNAs were isolated using the TRIzol Reagent. The results were analyzed by qRT-PCR. The experiment was performed 3 times.

### 2.10. Luciferase Assays

Cells were first cultured in a 24-well plate (1 × 10^4^ cells per well) for 2 d. They were then transfected with the indicated vectors using a Lipofectamine 3000 kit (Invitrogen) in accordance with the manufacturer protocols. After 2 d, the luciferase activities were measured using the Dual-Luciferase Reporter Assay System (Promega). The experiment was performed 3 times.

### 2.11. Chromatin Immunoprecipitation (ChIP) Assay

Infected CC cells were stained with 1% formaldehyde and lysed at room temperature for 10 min. The genome was fragmented to ~500 bp upon sonication. Antibodies were used to incubate the cell lysate for 12 hours. The lysate was subsequently incubated with Protein A Agarose/Salmon Sperm DNA (50% Slurry) beads for 6 hours. The results were analyzed by qRT-PCR assay.

### 2.12. Statistical Analysis

The GraphPad Prism software (GraphPad, USA) was used to calculate all data in this study. Statistical analysis was performed using two-tailed Student's *t*-test. *p* < 0.05 indicated a significant difference. Data in this study are presented using the mean ± square deviation (SD).

## 3. Results

### 3.1. Expression Characteristics of lncRNA SNHG15 in Cervical Cancer

**Figure 1:**
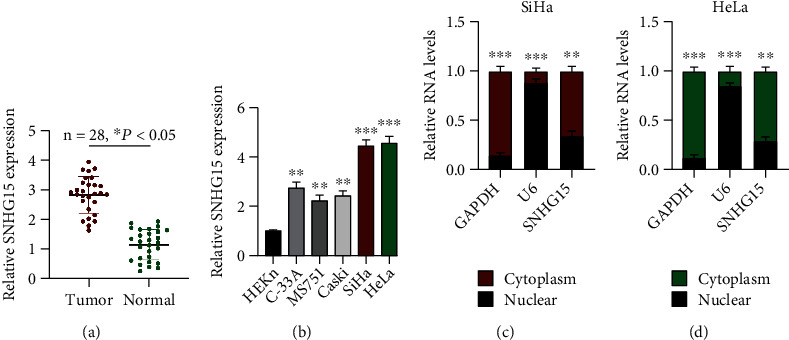
Expression characteristics of lncRNA SNHG15 in cervical cancer (CC). (a) lncRNA SNHG15 expression in CC tumor samples (*n* = 28) and adjacent normal samples (*n* = 28), measured by qRT-PCR. (b) lncRNA SNHG15 expression in CC cell lines, assessed by qRT-PCR. (c, d) lncRNA SNHG15 expression distribution in (c) SiHa and (d) HeLa cells, assessed using the cellular distribution assay. Data are presented as mean ± SD; ^∗^*p* < 0.5, ^∗∗^*p* < 0.01, and ^∗∗∗^*p* < 0.001.

To identify the possible function of lncRNA SNHG15 in CC progression, we first investigated the expression profile of lncRNA SNHG15 in CC. Twenty-eight pairs of CC tumor tissues and its comparative normal tissues were examined. lncRNA SNHG15 was noticeably upregulated in CC tumor tissues relative to that in normal tissues (Figure 1(a)). We further found that lncRNA SNHG15 was abundantly expressed in CC cell lines (Figure 1(b)) and mainly located in the cell cytoplasm (Figures 1(c) and 1(d)). On the basis of these combined findings, we hypothesized that lncRNA SNHG15 is involved in CC progression.

### 3.2. lncRNA SNHG15 Knockdown Attenuates CC Tumorigenesis and Chemoresistance *In Vitro*

**Figure 2:**
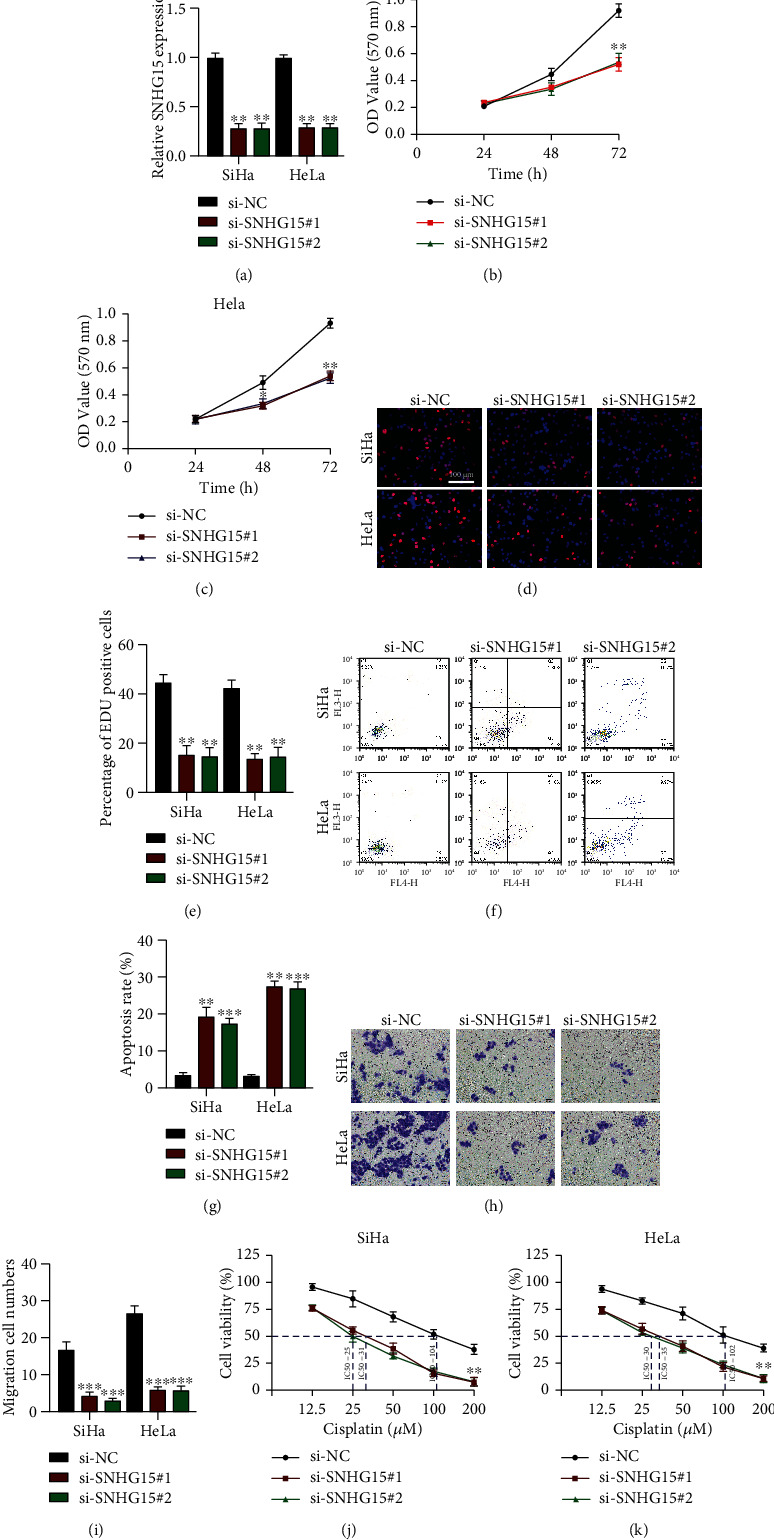
lncRNA SNHG15 knockdown attenuates cervical cancer (CC) tumorigenesis and chemoresistance *in vitro*. (a) lncRNA SNHG15 expression in CC cells after knockdown with two siRNAs, measured by qRT-PCR. (b, c) Cell proliferation in (b) SiHa and (c) HeLa cells, detected using the MTT assay. (d, e) Cell viabilities in (d) SiHa and HeLa cells, evaluated using the EdU assay; (e) analysis of results. (f) Cell apoptosis measured by flow cytometry assay and (g) calculation of the results. (h) Cell migration, assessed using the transwell migration experiment; (i) statistical analysis. (j) SiHa and (k) HeLa cells were treated with cisplatin at varying concentrations; chemoresistance of CC cells was evaluated using the MTT assay. Data are presented as mean ± SD; ^∗∗^*p* < 0.01 and ^∗∗∗^*p* < 0.001.

Given the abundant expression of lncRNA SNHG15 in CC tumor tissues and cells, we generated knockdown cell models of lncRNA SNHG15 to explore its cellular function (Figure 2(a)). We first examined the proliferation level of lncRNA SNHG15 knockdown cells by using the MTT assay (Figures 2(b) and 2(c)) and the EdU assay (Figures 2(d) and 2(e)) in SiHa and HeLa cells and found that lncRNA SNHG15 downregulation markedly suppressed CC cell proliferation. lncRNA SNHG15 downregulation significantly promoted the cell apoptosis rate (Figures 2(f) and 2(g)), as determined by flow cytometer assay. Moreover, lncRNA SNHG15 downregulation distinctly inhibited the CC cell migration, as determined using the transwell assay (Figures 2(h) and 2(i)). Notably, we examined whether lncRNA SNHG15 influences the resistance of CC cells to cisplatin, and lncRNA SNHG15 knockdown reduced the inhibitory concentration 50% (IC50) of SiHa and HeLa cells (Figures 2(j) and 2(k), respectively), respectively, indicating that lncRNA SNHG15 downregulation reduced the chemoresistance of CC cells to cisplatin. The results demonstrated that lncRNA SNHG15 knockdown inhibited cell proliferation, migration, and chemoresistance to cisplatin, as well as promoted cell apoptosis. Gain-of-function assays were also performed to confirm the functional role of lncRNA SNHG15 in CC progression (Supplementary Materials Figure S1); lncRNA SNHG15 overexpression promoted cell proliferation, migration, and chemoresistance to cisplatin but repressed cell apoptosis.

### 3.3. lncRNA SNHG15 Expression in CC Is Transcriptionally Regulated by SOX12

**Figure 3:**
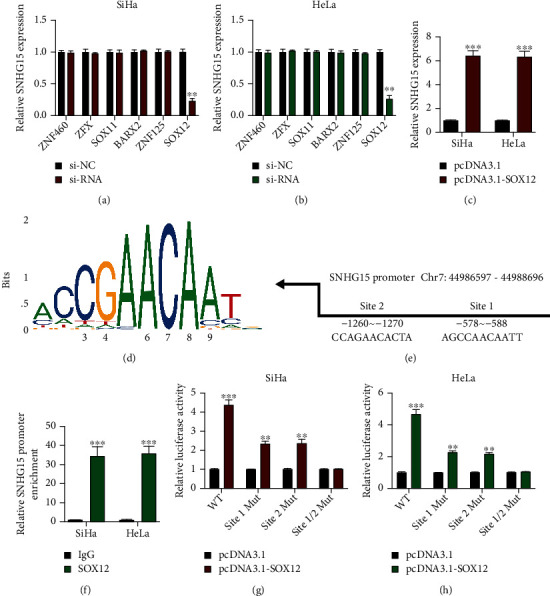
lncRNA SNHG15 expression in cervical cancer is transcriptionally regulated by SOX12. The upstream regulator of lncRNA SNHG15, predicted using the JASPAR (http://jaspar.genereg.net/) database. (a, b) SiHa and HeLa cells stably transfected with siRNAs; lncRNA SNHG15 expression, measured by qRT-PCR. (c) SiHa and HeLa cells transfected with pcDNA3.1-SOX12 and its normal control; lncRNA SNHG15 expression was assessed by qRT-PCR. (d) Binding motif of SOX12 predicted by JASPAR. (e) Putative binding sites for the lncRNA SNHG15 promoter, determined using JASPAR. (f) Binding possibility between SOX12 and the lncRNA SNHG15 promoter, assessed by ChIP assay. (g, h) Interaction between SOX12 and the lncRNA SNHG15 promoter in (g) SiHa and (h) HeLa cells, verified using the luciferase reporter gene assay. Data are presented as mean ± SD; ^∗∗^*p* < 0.01; ^∗∗∗^*p* < 0.001.

We investigated the upstream regulator of lncRNA SNHG15 in CC cells. Bioinformatics analysis was conducted, and 6 potential transcriptional regulators were predicted. We found that lncRNA SNHG15 expression was markedly decreased in CC cells under SRY-Box Transcription Factor 12 (SOX12) knockdown (Figures 3(a) and 3(b)) and increased in CC cells upon SOX12 overexpression (Figure 3(c)). The binding motif in SOX12 and the binding sites of the lncRNA SNHG15 promoter were acquired from the JASPAR database (Figures 3(e) and 3(e)). Chromatin immunoprecipitation (ChIP) assay results showed that the lncRNA SNHG15 promoter was pulled down by the SOX12 antibody in the SiHa and HeLa cells (Figure 3(f)). The interaction between SOX12 and the lncRNA SNHG15 promoter was verified by the luciferase reporter assay conducted on the SiHa and HeLa cells (Figures 3(g) and 3(h)). Combined, these findings indicated that SOX12 modulated lncRNA SNHG15 transcription in CC cells.

### 3.4. lncRNA SNHG15 Sponges miR-4735-3p

**Figure 4:**
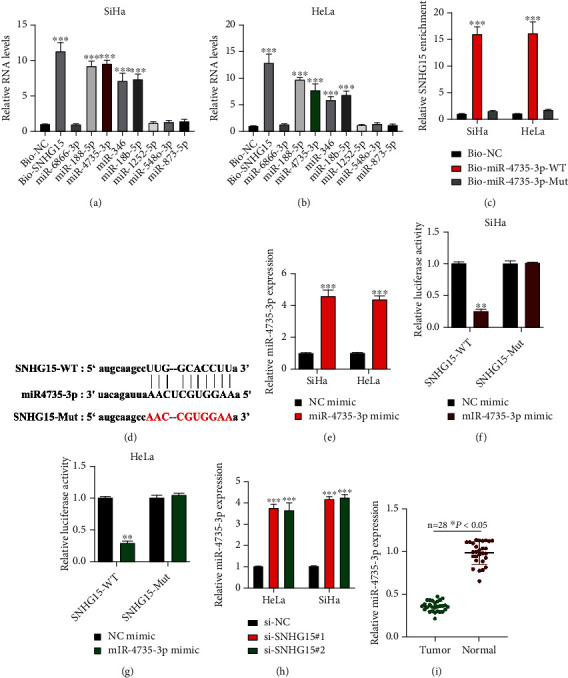
lncRNA SNHG15 sponges miR-4735-3p. Putative downstream targets for lncRNA SNHG15, predicted using the starBase (http://starbase.sysu.edu.cn/) dataset (CLIP Data: strict stringency (≥5)). (a, b) Potential miRNA targets for lncRNA SNHG15 in SiHa and HeLa cells, evaluated by biotinylated RNA pull-down; expression levels of putative miRNA targets in biotinylated probe bounds were measured by qRT-PCR. (c) Expression levels of lncRNA SNHG15 in Bio-miR-4735-3p-WT or Bio-miR-4735-3p-Mut bounds, measured by qRT-PCR. (d) Binding sites between lncRNA SNHG15 and miR-4735-3p, obtained using the starBase dataset. (e) Transfection efficiency of the miR-4735-3p mimic, evaluated by qRT-PCR. (f, g) Association between lncRNA SNHG15 and miR-4735-3p, assessed using the luciferase reporter gene assay in (f) SiHa and (g) HeLa. (h) miR-4735-3p expression in lncRNA SNHG15 knockdown cervical cancer (CC) cells, assessed by qRT-PCR. (i) miR-4735-3p expression in CC tumor samples (*n* = 28) and adjacent normal samples (*n* = 28), measured by qRT-PCR. Data are presented as mean ± SD; ^∗^*p* < 0.5; ^∗∗^*p* < 0.01; ^∗∗∗^*p* < 0.001.

The downstream target of lncRNA SNHG15 in CC cells was explored. The prediction from the starBase dataset showed 8 putative miRNA targets. Data from the biotinylated RNA pull-down assay revealed that 4 miRNA targets could potentially interact with lncRNA SNHG15 in CC cells (Figures 4(a) and 4(b)). Among the potential interactions, those between lncRNA SNHG15 and miR-188-5p [17, 18], miR-346 [19], and miR-18b-5p [20] have been previously verified. In the current study, we investigated the relation between lncRNA SNHG15 and miR-4735-3p in CC cells. Results from the RNA pull-down assay indicated that lncRNA SNHG15 could be pulled down by miR-4735-3p probes in SiHa and HeLa cells (Figure 4(c)). Binding sites between lncRNA SNHG15 and miR-4735-3p were revealed (Figure 4(d)), and the transfection efficiency of the miR-4735-3p mimic was assessed (Figure 4(e)). Results from the luciferase reporter assay suggested that lncRNA SNHG15 directly targeted miR-4735-3p in CC cells (Figures 4(f) and 4(g)). Moreover, miR-4735-3p expression in CC cells was upregulated under lncRNA SNHG15 knockdown (Figure 4(h)) and upregulated in CC tumors relative to that in normal samples (Figure 4(i)). These data revealed the miR-4735-3p as a downstream target for lncRNA SNHG15 in CC.

### 3.5. miR-4735-3p Modulates Tumorigenesis and Chemoresistance in Cervical Cancer

**Figure 5:**
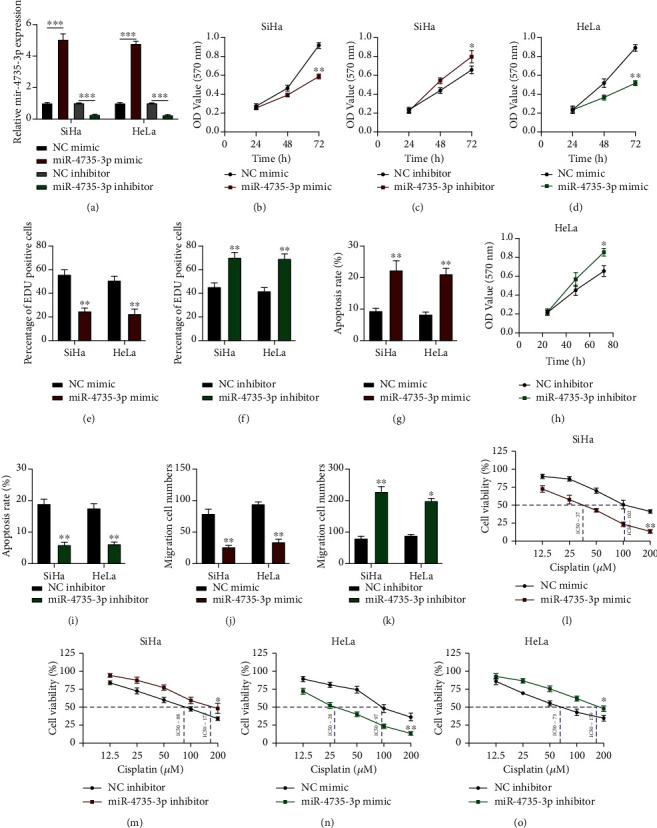
miR-4735-3p modulates tumorigenesis and chemoresistance in cervical cancer. (a) The gain- or loss-of-function of miR-4735-3p cell models was generated as indicated. (b–d) Cell proliferation of SiHa and HeLa cells upon miR-4735-3p dysregulation, detected using the MTT assay. (f, g) Cell proliferation levels of SiHa and HeLa cells upon miR-4735-3p (f) overexpression or (g) downregulation, evaluated using the EdU assay. (h, i) Cell apoptosis of SiHa and HeLa cells upon miR-4735-3p (h) overexpression or (i) downregulation, measured by flow cytometry. (j, k) Cell migration levels of SiHa and HeLa cells upon miR-4735-3p (j) overexpression or (k) downregulation, assessed using the transwell migration assay. (l–o) Chemoresistance of SiHa and HeLa cells upon miR-4735-3p dysregulation, detected using the MTT assay. Data are presented as mean ± SD, ^∗^*p* < 0.5; ^∗∗^*p* < 0.01; ^∗∗∗^*p* < 0.001.

We revealed miR-4735-3p as a target for lncRNA SNHG15 in CC cells; however, the function of miR-4735-3p remained unverified. Thus, we conducted a gain- or loss-of-function experiment on miR-4735-3p in CC cells. Cell models were generated as indicated (Figure 5(a)). CC cell proliferation was markedly suppressed by the miR-4735-3p mimic but promoted by the miR-4735-3p inhibitor, as determined using the MTT assay (Figures 5(b)–5(e)) and the EdU assay (Figures 5(f) and 5(g), Supplementary Materials Figures S2A–S2B). Flow cytometry results showed that the CC cell apoptosis rate was promoted by the miR-4735-3p mimic and inhibited by the miR-4735-3p inhibitor (Figures 5(h) and 5(i)). Transwell migration assay results suggested that CC cell migration was suppressed by the miR-4735 mimic but aggravated by the miR-4735-3p inhibitor (Figures 5(j) and 5(k), Supplementary Materials Figure S2C–S2D). For the same occurrence, the chemoresistance of CC cells to cisplatin was reduced by the miR-4735-3p mimic but aggravated by the miR-4735-3p inhibitor (Figures 5(l)–5(o)). Our results suggested that miR-4735-3p acted as a tumor suppressor in CC progression.

### 3.6. miR-4735-3p Targets to HIF1a

**Figure 6:**
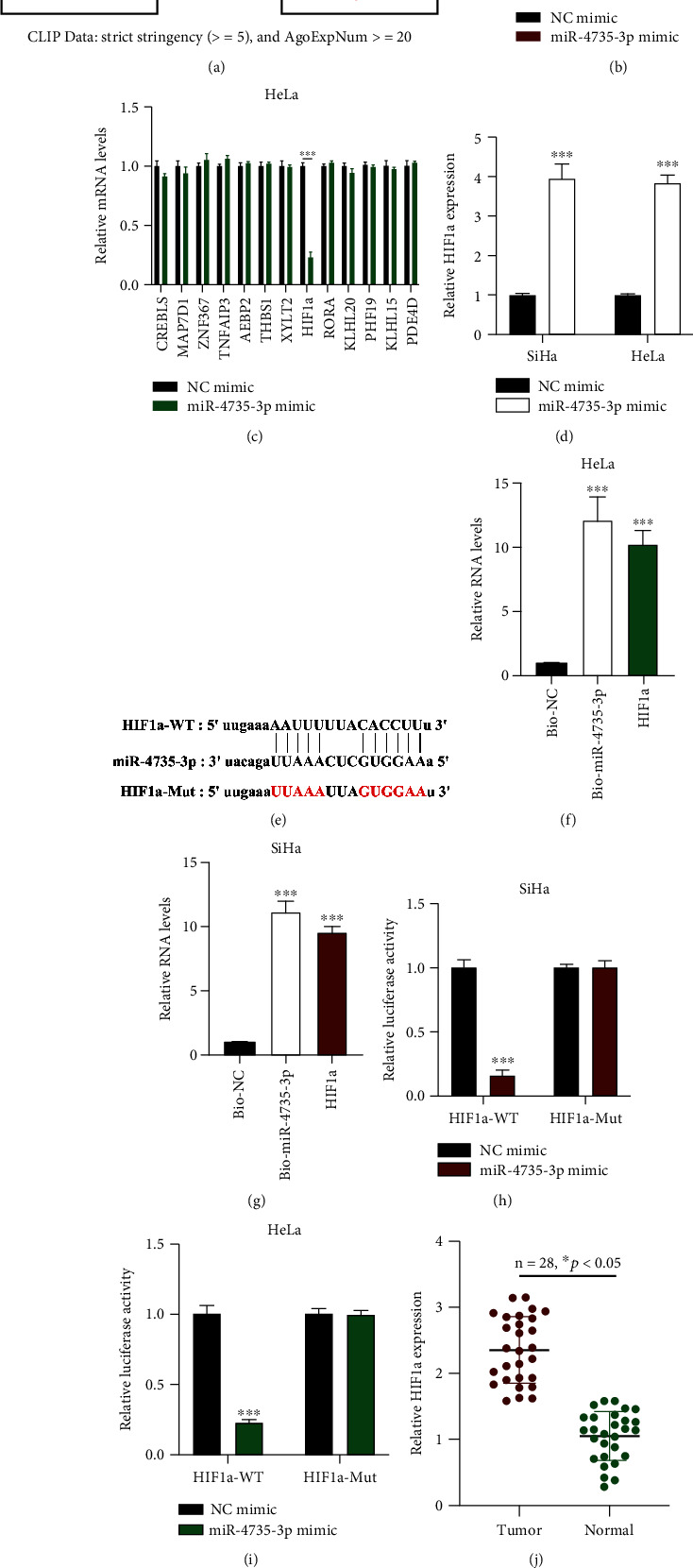
miR-4735-3p targets to HIF1a. (a) Putative downstream targets for miR-4735-3p, predicted by microT (http://www.microrna.gr/microT), miRmap (http://mirnamap.mbc.nctu.edu.tw/), PicTar (http://www.pictar.org/), and TargetScan (http://www.targetscan.org/) with CLIP Data: strict stringency (≥5) and AgoExpNum ≥ 20. (b, c) Putative mRNA targets in (b) SiHa and (c) HeLa cells upon miR-4735-3p overexpression were measured by qRT-PCR. (d) HIF1a expression in cervical cancer (CC) cells upon miR-4735-3p downregulation, measured by qRT-PCR. (e) Binding sites between HIF1a and miR-4735-3p. (f, g) Association between miR-4735-3p and HIF1a in (f) SiHa and (g) HeLa cells, evaluated by ChIP assay; analysis of results was by qRT-PCR. (h, i) Interaction between HIF1a and miR-4735-3p, assessed using the luciferase reporter gene assay in (h) SiHa and (i) HeLa. (j) HIF1a expression in CC tumor samples (*n* = 28) and adjacent normal samples (*n* = 28), measured by qRT-PCR. (k) HIF1a expression in 4 randomly chosen pairs of CC tissues, detected by Western blot. Data are presented as mean ± SD; ^∗^*p* < 0.5; ^∗∗∗^*p* < 0.001.

A total of 13 mRNA targets of miR-4735-3p were found by bioinformatics analysis (Figure 6(a)). We then found that HIF1a expression was decreased in miR-4735-3p overexpressed CC cells (Figures 6(b) and 6(c)) and increased in miR-4735-3p knockdown CC cells (Figure 6(d)), indicating that miR-4735-3p could be a potential target to HIF1a. The binding sites between miR-4735-3p and HIF1a were then obtained as indicated (Figure 6(e)). Data from the RNA pull-down assay showed that HIF1a was noticeably enriched in biotinylated miR-4735-3p probes in CC cells (Figures 6(f) and 6(g)). Meanwhile, luciferase reporter gene assay results confirmed that miR-4735-3p directly sponged to HIF1a in CC cells (Figures 6(h) and 6(i)). The interaction between miR-4735-3p and HIF1a was verified. HIF1a expression in CC tissues was measured; HIF1a expression in CC tumors was markedly higher than that in comparative normal tissues (Figures 6(j) and 6(k)).

### 3.7. lncRNA SNHG15 Regulates the Tumorigenesis and Chemoresistance of Cervical Cancer via the miR-4735-3p/HIF1a Pathway

**Figure 7:**
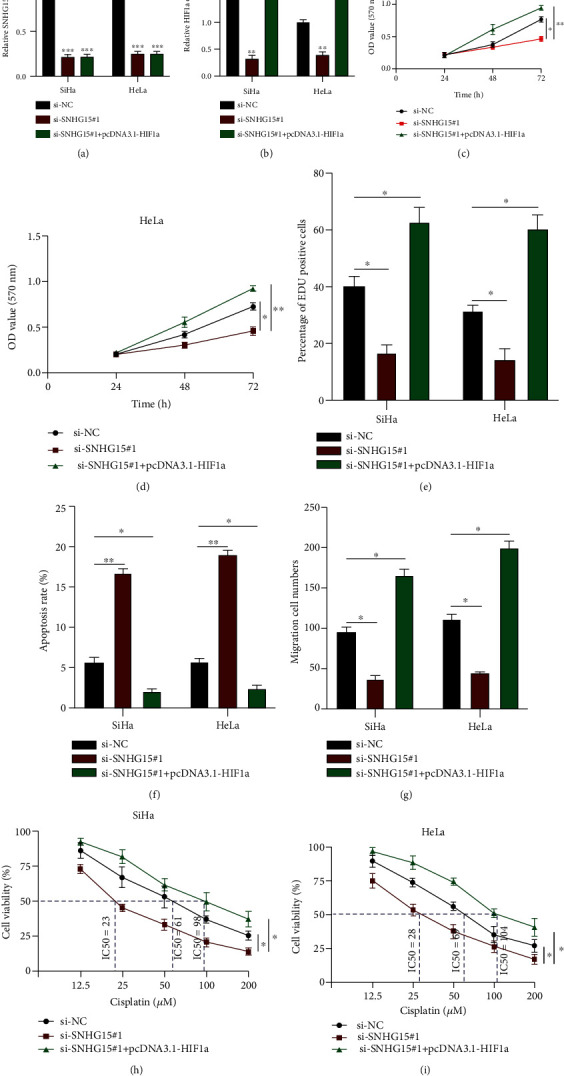
lncRNA SNHG15 regulates the tumorigenesis and chemoresistance of cervical cancer (CC) via the miR-4735-3p/HIF1a pathway. (a, b) Gain- or loss-of-function cell models were generated as indicated; assessment of transfection efficiencies. (c–e) Cell proliferation detected using the (c, d) MTT assay and the (e) EdU assay, respectively. (f) Cell apoptosis rate evaluated by flow cytometry. (g) Cell migration level assessed using the transwell migration assay. (h, i) SiHa and HeLa cells were treated with cisplatin at varying concentrations; the chemoresistance of CC cells was determined using the MTT assay. Data are presented as mean ± SD; ^∗^*p* < 0.5; ^∗∗^*p* < 0.01; ^∗∗∗^*p* < 0.001.

To further verify the molecular and cellular functions of lncRNA SNHG15 in CC progression, we generated cell models by transfecting si-NC, si-SNHG15#1, and si-SNHG15#1+pcDNA3.1-HIF1a into SiHa and HeLa cells, and lncRNA SNHG15 or HIF1a expression was detected (Figures 7(a) and 7(b)). HIF1a reversed the inhibitory effect of lncRNA SNHG15 knockdown on cell proliferation as detected using the MTT assay and the EdU assay (Figures 7(c)–7(e), Supplementary Materials Figure S3A). The cell apoptosis rate increased subsequent to lncRNA SNHG15 knockdown attenuated by HIF1a in CC cells (Figure 7(f)). Moreover, lncRNA SNHG15 knockdown suppressed cell migration level, and those occurrences were rescued by HIF1a in CC cells (Figure 7(g), Supplementary Materials Figure S3B). With regard to the chemoresistance of CC cells to cisplatin, lncRNA SNHG15 knockdown inhibited the IC50 value of SiHa and HeLa cells, but this occurrence was reversed by HIF1a (Figures 7(h) and 7(i)). Our results showed that lncRNA SNHG15 modulated HIF1a expression to regulate CC progression.

### 3.8. lncRNA SNHG15 Knockdown Suppresses CC Tumor Growth *In Vivo*

**Figure 8:**
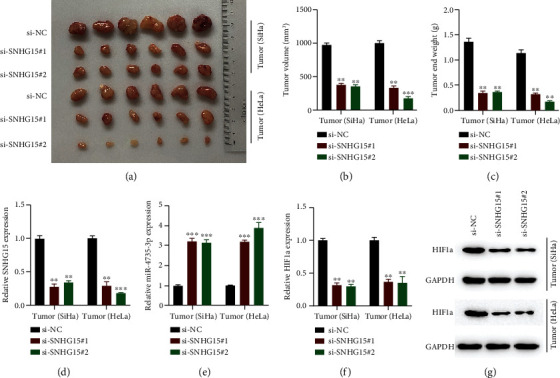
lncRNA SNHG15 knockdown suppresses tumor growth in cervical cancer *in vivo*. SiHa and HeLa cells stably transfected with si-NC, si-SNHG15#1, and si-SNHG15#2 were subcutaneously injected into the murine models (10^6^ cells/tumor: *n* = 6). (a) Representative image of xenograft tumors. (b) Tumor volume was recorded and calculated. (c) Tumor end weight was recorded. (d) lncRNA SNHG15 expression in tumors, measured by qRT-PCR. (e) miR-4735-3p expression determined by qRT-PCR. (f, g) HIF1a expression in resected tumor tissues, measured by qRT-PCR and Western blot analysis. Data are presented as mean ± SD; ^∗∗^*p* < 0.01; ^∗∗∗^*p* < 0.001.

The aforementioned experiments demonstrated the function of lncRNA SNHG15 *in vitro*. We generated mouse models to examine the role of lncRNA SNHG15 *in vivo*. lncRNA SNHG15 knockdown cells (10^6^ per murine) were subcutaneously injected into the mice, and after 4 weeks, tumors were collected. The representative image of xenograft tumors is presented in Figure 8(a). The tumor volume and the end weight were significantly inhibited by lncRNA NHG15 knockdown (Figures 8(b) and 8(c)). Further, the expression levels of SNHG15, miR-4735-3p, and HIF1a in lncRNA SNHG15 knockdown tumor tissues were measured (Figures 8(d)–8(g)), and *t* and the trends were consistent with our *in vitro* experiments. The results obtained suggest that lncRNA SNHG15 regulated tumor growth in murine models via the miR-4735-3p/HIF1a axis.

## 4. Discussion

The role of the lncRNA/miRNA/mRNA network in various biological progression has been elucidated in recent decades. As a major cause of cancer initiation and progression, multiple gene mutations have drawn wide scientific interests, and considerable effort has been exerted to elucidate the molecular mechanisms that contribute to tumorigenesis via the lncRNA/miRNA/mRNA network, such as bladder cancer, hepatocellular carcinoma, osteosarcoma, gastric cancer, oral cancer, and CC [21–26]. lncRNA-CTS regulates CC progression via the miR-505/ZEB2 axis [27], lncRNA TP73-AS1 modulates CC development via the miR-329-3p/ARF1 axis [28], lncRNA CAR10 promotes CC progression via the miR-125b-5p/PDPK1 pathway [29], and lncRNA DANCR aggravates CC development via the miR-335-5p/ROCK1 axis [30]. Despite the accumulating reports on the lncRNA/miRNA/mRNA network globally, the main mechanism underlying gene dysregulation in cancer development has yet to be clarified.

In this study, we clarified elucidated that lncRNA SNHG15 was highly expressed in CC tissues and cells. Downregulated lncRNA SNHG15 attenuated tumorigenic properties *in vitro* and CC tumor growth *in vivo*. lncRNA SNHG15 dysregulation also influenced the chemoresistance of CC cells to cisplatin. Cisplatin belongs to the alkylating antineoplastic agent and is used in the clinical management of several malignancies, including CC [31–36]. With the wide clinical application of cisplatin, its side effects are been revealed, such as nausea, vomiting, kidney damage, hearing loss, and cell chemoresistance [37]. Understanding the molecular mechanisms underlying chemoresistance, among its side effects, is crucial to improving the therapy efficiency of cisplatin. The results of this study indicate that lncRNA SNHG15 downregulation inhibited the IC50 value of CC cells upon cisplatin treatment.

Further, for the upstream regulator of lncRNA SNHG15, we found that lncRNA SNHG15 expression in CC cells was transcriptionally regulated by SOX12. For the downstream factors of lncRNA SNHG15, our results demonstrated that lncRNA SNHG15 promoted HIF1a expression via sponging of miR-4735-3p in CC cells. Our biological experiments revealed that lncRNA SNHG15 promotes the tumorigenic properties and chemoresistance of CC cells via the miR-4735-3p/HIF1a axis. Meanwhile, this study has several limitations. First, more human samples are required to confirm the clinical significance of lncRNA SNHG15 in CC tumors. Second, a larger scale of samples than the one used in this study is needed to examine the correlation of expression between lncRNA SNHG15, miR-4735-3p, and HIF1a in CC tumors. Finally, the specific molecular relation between HIF1a and CC cellular progression requires an in-depth investigation, such as determining the involved pathway.

## 5. Conclusion

In summary, our study elucidates the expression and biological function of lncRNA SNHG15 in CC. This study also indicates that SOX12 could transcriptionally regulate lncRNA SNHG15 expression in CC cells. lncRNA SNHG15 promotes CC progression by regulating the miR-4735-3p/HIF1a axis, which could be a potential target for the diagnosis of CC or clinical intervention for the disease in the future.

## Data Availability

The research data used to support the findings of this study are included within the article.

## Supplementary Materials

Supplementary Materials Figure S1: lncRNA SNHG15 overexpression aggravates CC tumorigenesis and chemoresistance in vitro. (A) lncRNA SNHG15 level was measured by qRT-PCR in CC cells. (B, C) Cell proliferation was detected by MTT assay in SiHa (B) and HeLa cells (C). (D, E): EdU assay was applied to evaluate cell viabilities in SiHa and HeLa cells (D), and results were analyzed as indicated (E). (F, G) Cell apoptosis rate was measured by flow cytometer assay (F), and results were calculated as indicated (G). (H, I) Transwell migration experiment was performed to assess cell migration level (H), and statistical analysis was presented (I). (J, K) SiHa (J) and HeLa (K) cells were treated with cisplatin at various concentrations as indicated, and MTT assay was applied to evaluate the chemoresistance of CC cells. Data were presented as mean ± SD; ^∗∗^*p* < 0.01 and ^∗∗∗^*p* < 0.001. Figure S2: (A, B) EdU assay was applied to evaluate cell proliferation of SiHa and HeLa cells upon miR-4735-3p overexpression (A) or downregulation (B). (C, D) Transwell migration assay was conducted to assess cell migration level of SiHa and HeLa cells upon miR-4735-3p overexpression (C) or downregulation (D). Figure S3: (A) Cell proliferation was detected by EdU assay as indicated. (B) Cell migration level was assessed by transwell migration assay.
