# Supplementary material for: lncRNA SNHG15 Induced by SOX12 Promotes the Tumorigenic Properties and Chemoresistance in Cervical Cancer via the miR-4735-3p/HIF1a Pathway
Source: Oxid Med Cell Longev. 2022 Jan 12;2022:8548461. doi: 10.1155/2022/8548461 (PMC8769851; doi:10.1155/2022/8548461)
Supplement: Supplementary Materials — Figure S1: lncRNA SNHG15 overexpression aggravates CC tumorigenesis and chemoresistance in vitro. (A) lncRNA SNHG15 level was measured by qRT-PCR in CC cells. (B, C) Cell proliferation was detected by MTT assay in SiHa (B) and HeLa cells (C). (D, E): EdU assay was applied to evaluate cell viabilities in SiHa and HeLa cells (D), and results were analyzed as indicated (E). (F, G) Cell apoptosis rate was measured by flow cytometer assay (F), and results were calculated as indicated (G). (H, I) Transwell migration experiment was performed to assess cell migration level (H), and statistical analysis was presented (I). (J, K) SiHa (J) and HeLa (K) cells were treated with cisplatin at various concentrations as indicated, and MTT assay was applied to evaluate the chemoresistance of CC cells. Data were presented as mean ± SD; ∗∗p < 0.01 and ∗∗∗p < 0.001. Figure S2: (A, B) EdU assay was applied to evaluate cell proliferation of SiHa and HeLa cells upon miR-4735-3p overexpression (A) or downregulation (B). (C, D) Transwell migration assay was conducted to assess cell migration level of SiHa and HeLa cells upon miR-4735-3p overexpression (C) or downregulation (D). Figure S3: (A) Cell proliferation was detected by EdU assay as indicated. (B) Cell migration level was assessed by transwell migration assay. [file 8548461.f1.docx]

Supplementary Figures (S1, S2, and S3)

Figure. S1. LncRNA SNHG15 overexpression aggravates CC tumorigenesis and chemoresistance *in vitro*. A: LncRNA SNHG15 level was measured by qRT-PCR in CC cells. B and C: Cell proliferation was detected by MTT assay in SiHa (B) and HeLa cells (C). D and E: EDU assay was applied to evaluate cell viabilities in SiHa and HeLa cells (D), results were analyzed as indicated (E). F and G: Cell apoptosis rate was measured by Flow cytometer assay (F), results were calculated as indicated (G). H and I: Transwell migration experiment was performed to assess cell migration level (H), statistical analysis was presented (I). J and K: SiHa (J) and HeLa (K) cells were treated with cisplatin at various concentrations as indicated, MTT assay was applied to evaluate the chemoresistance of CC cells. Data were presented as mean ± SD, ***p* < 0.01, ****p* < 0.001.

Supplementary Figure S2.

A-B: EDU assay was applied to evaluate cell proliferation of SiHa and HeLa cells upon miR-4735-3p overexpression (A) or downregulation (B). C-D: Transwell migration assay was conducted to assess cell migration level of SiHa and HeLa cells upon miR-4735-3p overexpression (C) or downregulation (D).

Supplementary Figure S3.

A: Cell proliferation was detected by EDU assay as indicated. B: Cell migration level was assessed by Transwell migration assay.
